# The Impact of Complexity on Methods and Findings in Psychological Science

**DOI:** 10.3389/fpsyg.2020.580111

**Published:** 2021-01-21

**Authors:** David M. Sanbonmatsu, Emily H. Cooley, Jonathan E. Butner

**Affiliations:** Department of Psychology, University of Utah, Salt Lake City, UT, United States

**Keywords:** complexity, scientific rigor, measurement, reproduction, methods

## Abstract

The study of human behavior is severely hampered by logistical problems, ethical and legal constraints, and funding shortfalls. However, the biggest difficulty of conducting social and behavioral research is the extraordinary complexity of the study phenomena. In this article, we review the impact of complexity on research design, hypothesis testing, measurement, data analyses, reproducibility, and the communication of findings in psychological science. The systematic investigation of the world often requires different approaches because of the variability in complexity. Confirmatory testing, multi-factorial designs, survey methods, large samples, and modeling are frequently needed to study complex social and behavioral topics. Complexity impedes the measurement of general constructs, the reproducibility of results and scientific reporting, and the general rigor of research. Many of the benchmarks established by classic work in physical science are not attainable in studies of more complex phenomena. Consequently, the standards used to evaluate scientific research should be tethered to the complexity of the study topic.

## Introduction

For decades, researchers in the physical sciences have implored scientists in other fields to be like them. The assumption underlying this advice has been that disciplines such as economics and geology would advance more rapidly if they adopted the practices and standards of fields such as chemistry and astronomy. While much of this patronizing guidance has ebbed, the thinking still persists. In 2005, the journal *Nature* published an editorial entitled “In praise of soft science” featuring the lead: “‘Hard’ scientists should stop looking down their noses at social scientists, and instead share methods that could help them address pressing societal problems” (p. 1003). The bottom line of the condescending editorial was that social scientists are studying important topics; they just need help from the real sciences to progress.

The attitude of many researchers in the “hard” sciences is understandable, given the vast differences in the achievements of fields. The truth is that the social and behavioral sciences have made substantially less progress than other disciplines in developing precise quantitative theories and efficacious interventions and treatments. The contrasts in success and stature have motivated generations of researchers to strive to be like physicists and chemists. In fact, for over half a century, many social and behavioral scientists dutifully adopted the methods and approaches of the physical sciences.

Scholars have long believed that as the social and behavioral sciences matured, they would become more like their highly regarded counterparts. The assumption here has been that with the passage of time, the research procedures, designs, and measures in disciplines such as psychology and sociology would become more rigorous and refined. This, in turn, would lead to the establishment of precise theories and laws on par with those of physics and astronomy. However, as greater effort and resources have been invested in the study of behavior and groups, research methodologies have tended to become more varied and diverse. If anything, the social and behavioral sciences have become increasingly dissimilar from the “hard” sciences as they have matured.

## Science Is Not Always the Same

Scientists across disciplines share the same basic scholarly aspirations; they seek to describe and explain the world, to predict important events, and to develop applications that benefit their communities. While the general goals of all scientists are largely the same, the specific manner in which research is conducted varies substantially from discipline to discipline. This variability is most apparent in comparisons between the social and behavioral sciences, whose practices have been labeled “soft,” and the natural sciences, whose practices have been labeled “hard” (e.g., Storer, 1967).

There are many reasons why science is often conducted differently by social and behavior researchers. The procedures, measures, and treatments used in the social and behavioral studies are severely restricted by the legal and institutional regulations, moral concerns, and ethical guidelines governing human research. In addition, the scope, depth, and technology of studies have been limited by the historically low levels of funding allocated to the social and behavioral sciences. Research is further hampered by the tendency for humans to behave differently when they perceive that they are being observed and studied (e.g., Orne, 1962; Levitt and List, 2011). However, we believe that the primary contributor to disciplinary differences in the scientific enterprise is the variability in the complexity of the topics that are studied. Social and behavioral scientists generally investigate more complex phenomena than natural scientists. From decades of research, they have learned that traditional scientific approaches are not always well suited for the examination of challenging topics, such as the dynamics of organizations and the machinations of the mind. Researchers in fields, such as psychology, anthropology, and sociology, have found it necessary to develop new methods and approaches to study phenomena that are lacking in regularity and predictability.

Prior research (e.g., Cole, 1983; Simonton, 2004; Fanelli and Glänzel, 2013) has uncovered many important differences in research practices between disciplines in the context of investigating Comte’s (1855) hierarchy of sciences. Comte postulated that the sciences could be ordered in a hierarchy of increasing complexity and dependency and decreasing generality beginning with astronomy followed by physics, chemistry, biology, and sociology. Simonton (2004, 2015), Fanelli and Glänzel (2013), and other scholars have drawn from numerous sources, including publishing trends, surveys, and citations, to show that fields vary in generality, dependency, and complexity in a manner consistent with the rankings postulated by Comte.

While the research on Comte’s (1855) hierarchy has revealed many important disciplinary differences in scientific practice, we believe that the complexity of a research topic has much greater impact than has been previously recognized. In our view, complexity affects almost every facet of the scientific enterprise from research design to measurement to statistical analysis to scientific reporting. In this paper, we examine how the practice of science in psychology has been shaped by the complexity of the study phenomena. While the focus is psychology and other social and behavioral fields, we believe that our observations hold true for studies of complex phenomena in other scientific disciplines.

Our paper does not review the enormous literature on complexity in science or present new insights about the workings of complex systems. The paper also does not present a comprehensive review of all of the ways in which psychological research deviates from more traditional methods. Rather, we focus on the most central practices that are affected by the complexity of the study phenomena. In particular, we examine the impact of complexity on the design of studies, testing of hypotheses, measurement of variables, reporting of results, reproduction of findings, and general rigor in psychology.

## The Impact of Complexity on Theory Development

There is no consensus for a singular definition of “complexity” in science (Zuchowski, 2012; Fanelli and Glänzel, 2013). For purposes of this paper, complexity is defined by emergent properties, processes, and the behavior that are not reducible to lower level mechanisms or the workings of the individual parts (e.g., Van Regenmortel, 2004; Mazzocchi, 2008). Complexity is further defined by the number of components of a system, and the number and non-linearity of the connections or interrelations between the components. While emergent phenomena are commonly stable and measurable (e.g., Bedau and Humphreys, 2008; Taylor, 2009), complex systems are often mutable and change as a function of the interactions between their components and encounters with the broader environment. Thus, they have hysteresis, that is, their current state is dependent on their history.

Developing precise theories of the countless components and relations that often characterize complex systems is a formidable task. Sanbonmatsu and Johnston (2019) suggest that, as the phenomena of study become more complex, the development of theory is impeded by an increasing tradeoff between generality and precision. Scientists attempt to develop general theories that are comprehensive yet parsimonious. Limiting the number of terms or statements of a theory is important, of course, because it facilitates explanation, understanding, and communication. However, when the study topic is complex, the causes and moderators of important effects are more numerous and much less invariant. To represent this variability accurately, the descriptions of the causal relations and definitions of broad constructs have to be abstract and imprecise. Because of this, many theories in psychology and other social and behavioral sciences are only vaguely true. They are often presented in such qualitative terms that they are almost impossible to falsify.

While general theories may explain complex phenomena, they commonly lack the specificity to make exacting predictions. To increase precision, scientists have to focus on proximal determinants and develop applied theories and models that are specific to particular contexts and entities. Although these theories and models facilitate the development of applications and interventions, they tend to lack any semblance of generality.

Understanding the impact of complexity on theory development is important to our examination of disciplinary differences in scientific practice because theory drives research. Theory determines to a large degree the hypotheses that are tested, the constructs that are measured, and the general rigor of research. Complexity often influences methods by shaping the specificity of the theories and hypotheses that guide research. As we discuss, many of the challenges and problems characterizing research in psychological science and other disciplines are rooted in the difficulties of conceptualizing complex phenomena.

## Moving Target

The phenomena of interest in physics are truly general. The phenomena that sociologists attempt to understand frequently change faster than we are able to adequately describe them…. In sociology, by the time any theory is developed that might explain a particular phenomenon, it is possible that the phenomenon and the factors causing it will have changed. In short, sociologists are shooting at a moving target – a target that frequently has changed or disappeared by the time the bullet arrives (Cole, 1994, p. 138–139).

Basic researchers regard science as the search for eternal truths about the universe. However, there are obvious contrasts between disciplines and fields in the invariance of the causal relations that are studied and reported. One of the most fundamental differences between the biological, social, and behavioral sciences and the physical sciences is the “mutability” of the study phenomena. As Cole (1994) suggests in the quote above, researchers in complex fields are much more likely to study moving targets; that is, they are much more likely to investigate processes, behaviors, properties, and entities that change over time.

There are many mechanisms through which traits and behaviors “mutate.” Most fundamentally, alterations in the DNA sequence of genes may arise as a result of errors in the replication process. Environmental pressures, aging, or changes in health can also lead to epigenetic variations in the expression of genes that shape the behavior of organisms.

Causal relations may be altered through more social mechanisms. Changes in the structures, ideologies, laws, and norms of societies can dramatically alter peoples’ beliefs and actions. Broader changes in ecosystems, physical environments, and technological developments similarly impact how societies and individuals function. The patterns of responding also change through experience and learning. Some of these alterations are fostered by research. Unlike physical phenomena, human activity is directly impacted by the communication of scientific findings. As Gergen (1973, p. 313) stated,

“Herein lies a fundamental difference between the natural and social sciences. In the former, the scientist cannot typically communicate his knowledge to the subjects of his study such that their behavioral dispositions are modified. In the social sciences such communications can have a vital impact on behavior.”

The tendency for individual and group behavior to “mutate” adds another level of complexity to social and behavior science. Not only are the targets of research difficult to pinpoint, they are constantly moving. Moreover, just as scientists begin to track the target, their own research sometimes causes it to change direction.

## Confirmatory Hypothesis Testing in Psychological Science

Popper (1959) believed that science progresses primarily through falsification. He argued that, while theories can never be conclusively verified, they can be dismissed by a single disconfirming observation. In his view, the best scientific theories and hypotheses are falsifiable. In a related vein, Platt (1964) argued that, for “strong inference,” experiments need to eliminate less viable hypotheses in crucial tests. However, many philosophers and scientists have argued that scientific theories are based more on corroborations than falsifications (e.g., Ladyman, 2002). Disconfirmations are commonly dismissed because of misassumptions in the “auxiliary hypotheses” (Quine, 1953; Duhem, 1962). Moreover, theories are typically adjusted to accommodate disconfirming findings (Lakatos, 1978). Similarly, crucial tests often fall short because hypotheses are conditional and modifiable (O’Donohue and Buchanan, 2001; Davis, 2006).

Some researchers (e.g., Meehl, 1978) have argued that psychological science needs to utilize more of the falsification strategy prescribed by Popper (1959) to progress. However, the complexity of social and behavioral phenomena often precludes the development and testing of falsifiable theories.

### The Diagnosticity of Confirmatory vs. Disconfirmatory Evidence Depends on the Test Hypothesis

Sanbonmatsu et al. (2005) argued that the appropriateness of a confirmatory vs. disconfirmatory strategy depends on the type of hypothesis under investigation (for purposes of this paper, “confirmatory” and “disconfirmatory” strategies are equated with “positive” and “negative” searches, respectively; see Klayman and Ha, 1987). Hypotheses specify the proportion of instances that are characterized by a particular relation or effect. At the broadest level, hypotheses are either absolute in presuming that a particular relation is always present or absent or non-absolute in presuming that a relation is sometimes present or absent. The diagnosticity of evidence depends on the hypothesized frequency of the test relation. Diagnosticity can be defined in terms of the degree to which the information distinguishes the test hypothesis from its complement (e.g., Fischhoff and Beyth-Marom, 1983). In tests of absolute or universal hypotheses, disconfirmations have considerably greater diagnostic value than confirmations. A confirming observation is possible not only when an absolute hypothesis is true but also when it is false. However, a disconfirming instance is not possible when a universal hypothesis is true. This, of course, is in keeping with Popper’s (1959) analysis of the utility of falsification in science. In contrast, confirmations are much more diagnostic than disconfirmations in tests of the non-absolute hypothesis that an effect occurs sometimes or in some conditions. While a confirming observation is possible only when a non-absolute hypothesis is true, a disconfirming observation is possible when a non-absolute hypothesis is both true and false.

People are generally cognizant that the diagnosticity of confirming vs. disconfirming evidence depends on the universality of the hypothesized relation. Research has shown that both laypersons (Sanbonmatsu et al., 2005) and psychological scientists (Sanbonmatsu et al., 2015) are more likely to use a confirmatory approach in the testing of non-absolute hypotheses and a disconfirmatory approach in the testing of universal hypotheses.

### Social and Behavioral Studies Are More Likely to Take a Confirmatory Approach Because of Complexity

The diagnosticity of disconfirmatory vs. confirmatory evidence varies as a function of the complexity of the study phenomena, and, hence, scientific discipline. When the subject matter is complex, there are more numerous causes, interactions, emergent processes, and non-linear relations. Because of this, the hypothesized relations that are tested are typically far from universal. Consequently, confirmations of hypotheses about complex phenomena are generally much more informative than disconfirmations. In contrast, disconfirmations are often more informative than confirmations in studies of simple phenomena because the hypothesis that are tested are often universal or near universal.

Theories in the natural sciences tend to be more falsifiable and, hence, “better” than theories in the social and behavioral sciences because the study phenomena are generally simpler and more invariant. The hypotheses that are generated in fields such as molecular biology are more likely to be absolute or near absolute and more subject to falsification. Thus, while confirmatory testing predominates all fields, disconfirmatory studies and “crucial tests” (Platt, 1964) may be conducted more frequently in the natural sciences than in the social and behavioral sciences.

Because of the complexity of social and behavioral phenomena, psychologists tend to use a confirmatory approach in their studies (Uchino et al., 2010). Evidence suggests that they are more apt to seek confirmation because the hypotheses they generate and test are generally non-universal (Sanbonmatsu et al., 2015). Disconfirmations are also less meaningful in the social and behavioral sciences because of the widespread methodological difficulties surrounding the study of complex phenomena. Research studies that fail to support the test hypothesis are frequently dismissed because of the shortcomings in the methods. As we will discuss later, studies in the social and behavioral sciences are commonly saddled with imprecise measures, weak manipulations, and a general lack of rigor because of the complexity of the study phenomena. These methodological issues undermine the theoretical conclusions that can be drawn from disconfirmatory findings, and hence, the extent to which they are publishable. We speculate that there are stronger norms against publishing negative results in fields such as psychology precisely because disconfirmations are generally less informative.

Evidence that the publishing of positive findings is more common in the social and behavioral sciences than in other fields was provided in an archival study by Fanelli (2010). The study analyzed over two thousand papers from numerous disciplines in which the testing of a hypothesis was reported. Positive results were reported with much greater frequency in social and behavioral science papers (including business) than in physical science and space science articles. While there are undoubtedly a number of contributing factors, we believe that the differences in the reporting of positive results across disciplines stem primarily from the lower informativeness of negative findings in fields such as psychology and sociology.

Social and behavioral scientists have been criticized for decades because of the lack of falsifiability of their theories (e.g., Popper, 1959). However, it seems unlikely that social and behavioral theories and hypotheses are less universal and, hence, less falsifiable because researchers are incapable of discovering patterns that are more uniform and general. It is equally incredulous that researchers would deliberately opt for more nuanced and convoluted theories over more parsimonious, general, and predictive conceptualizations of the world. The complex phenomena studied in the social and behavioral sciences simply do not lend themselves to the development of falsifiable theories.

## Disciplinary Differences in Design, Analyses, and Reporting

One of the most significant contributors to greater methodological diversity in science is the complexity of the study phenomena. In this section of the paper, we review the impact of complexity on research design, sampling, analyses, and reporting in psychology and other social and behavioral sciences. As we discuss, the numerousness and variability of the causal relations, emergent processes, and weak and inconsistent effects characteristic of complex phenomena often necessitate non-traditional methods.

### Research Design

To begin to understand complex social and behavioral phenomena, researchers often have to assess the role of multiple interactive causes and processes (e.g., Stanovich, 2019). Consequently, simple experiments often will not do (e.g., Cronbach, 1957, 1975). We speculate that experiments in psychological science are much more apt to be multi-factorial than experiments in the natural sciences. Psychologists may also be more likely to conduct a series of interrelated experiments to test potential determinants, moderators, and underlying processes. Indeed, in some of the more prominent journals in the field, papers are not publishable unless the reported findings entail this level of scope.

One of the major difficulties of studying the complex phenomena is the numerousness of the relevant variables (e.g., Meehl, 1978). Often, there are too many potential causes of complex phenomena to experimentally manipulate. Consequently, observational and survey methods are frequently utilized because they enable researchers to quickly and economically measure large swaths of variables and interrelations. The numerosity of variables also makes statistical analyses more challenging and creates potential pitfalls such as the omitted variable bias in regression.

To more fully capture the nuances of complex phenomena and the natural settings in which they are grounded, social and behavioral scientists conduct qualitative studies (e.g., Henwood and Pidgeon, 1992). Qualitative research often better captures the individual perspectives that shape behavior and is generally more sensitive to the context in which the causal effects occur than experimental designs. Case studies have been the important starting point for theory development and hypothesis generation in clinical neuropsychology (e.g., Damasio, 1994).

### Sampling

The tremendous variability of the components of many complex systems necessitates careful sampling. This is particularly true in social and behavioral research because every person is different. While testing the properties of a particular chemical compound generally does not require a sampling plan, the selection of an appropriate cohort is crucial to the external validity of a psychology study. Unfortunately, the logistics of obtaining a sample that is representative of all of humanity are so daunting and overwhelming that the field generally resorts to convenience and rationalization. The most common justification for not obtaining a representative sample is that psychologists are studying “basic processes” that are largely the same across persons. However, when studies fail to replicate because of sampling and other “random” sources of variability (e.g., Open Science Collaboration, 2015; Stanley et al., 2018), it becomes all too apparent that the processes and effects being investigated are not “basic” and universal and that convenience samples are not justifiable on conceptual grounds.

While most discussions of sampling focus on the selection of persons, the reality is that most of the behaviors and processes that are investigated in fields such as psychology and economics are affected by other study characteristics, such as the physical environment, the immediate social situation, culture, time, and operationalizations of the independent and dependent measures (Fiedler, 2011). However, because of the impossibility of constructing studies that are representative with respect to all facets of the procedures and context, researchers also resort to convenience in sampling these critical components. Studies are typically limited to one physical environment, one manipulation, one cultural setting, and one of every other important study element. Obviously, sampling is less of an issue in the physical sciences because the entities and causal relations that are investigated are often more uniform across time and place.

### Statistical Analyses

Statistics utilized across science are all basically the same. However, it is common to attempt to address many of the issues inherent in the study of complex topics through varying statistical techniques. For example, measurement issues are commonly modeled through latent variables within structural equation modeling as a means to account for measurement imprecision (Bollen, 1989); random effects are integral to mixed models as a way to allow for different effects from different people (Raudenbush and Bryk, 2002); and integrative data analysis seeks to expand on meta-analytic principles by simultaneously analyzing multiple data sets as a way to bridge replication and sampling issues (Curran and Hussong, 2009). So, it is the very issues discussed herein that frequently relate to disciplinary differences in the use of statistics. Strict experimentation with strong effects and clean measurement, for example, enables simpler statistical approaches.

Statistics play a central role in psychology when considering causal claims. The social and behavioral sciences heavily draw from Rubin’s (1974) causal model for experimentation. Random assignments, within subjects designs, and comparisons based on having identical units are all attempts to resolve causal issues. However, in the social and behavioral sciences, it is not always easy to apply Rubin’s model. For example, the model only applies to causes that can be manipulated, which cannot always be done or done ethically. Psychology’s reliance on quasi-experimentation and non-experimentation have instead relied on statistical associations in conjunction with the methodology to make up for the difference (Berk, 1988). Granger’s (1988) causal arguments rely on our ability to statistically control for other possible explanations. However, under complexity, linear and even non-linear associations that are estimated statistically may fail to meet Granger’s criteria (Sugihara et al., 2012), suggesting that causal arguments themselves may be difficult to claim.

### Scientific Reporting

Research suggests that dissertation abstracts and texts are longer in the social and behavioral sciences than in the physical sciences (Ashar and Shapiro, 1990, Table 2, p. 131). Similarly, Fanelli and Glänzel (2013) showed that as research fields get “softer,” the page length of articles tends to increase. Scientific articles may be longer in more complex disciplines because there is typically more to describe and explain. For example, in social and behavioral science papers, there are often more study hypotheses to justify and more measures to describe. Moreover, because of a less standardization, the procedures often require greater detailing. Results sections commonly go on and on because there are so many analyses to report. Finally, discussions are often lengthy because of inconsistencies in the data and discrepancies with the prevailing theory.

Findings are often reported differently in the social and behavioral sciences than in less complex disciplines, such as physics and molecular biology, because of the messiness of the results. Studies of what Simonton (2004) calls “graph prominence” have shown that articles in the natural sciences are much more likely to the results of the present study in graphs than articles in the social and behavioral sciences (Cleveland, 1984; Smith et al., 2000). Simonton (2015, p. 340) explains:

In the physical sciences, and to a slightly lesser extent the biological sciences, the results tend to be so clean, and the effect sizes so large, that the findings can easily be depicted in visual form. The error bars around a fitted curve are small, even trivial. By comparison, psychology and especially sociology deal with phenomena so complex that the results cannot be so simply portrayed. Hence, the findings may have to be presented in statistical tables, with the number of asterisks deceptively indicating importance (Meehl, 1978).

The imprecision of theories and the variability of findings in more complex fields also affects verbal presentations of research findings. Schachter et al. (1991) showed that undergraduate classroom lectures in the social and behavioral sciences and humanities were characterized by more frequent pauses (“uh,” “er,” and “um”) than similar lectures in the natural sciences. The variation in delivery was due to differences in content rather than skill as speech did not vary in disfluency when lecturers were interviewed about a common subject (teaching). Fluency is often lacking in the presentation of social and behavioral research because theories and findings are more complicated and require greater qualification. Psychologists routinely have to use phrases, such as “often,” “sometimes,” and “tends to,” that limits the generality and the strength of their statements because of the variability of the described relations and effects (Sanbonmatsu and Johnston, 2019).

## “Inadequate” Measurement

I should like to venture the judgment that it is inadequate measurement, more than inadequate concept or hypothesis, that has plagued social researchers and prevented fuller explanations of the variances with which they are confounded (Hauser, 1969, p. 129).

The measurement of theoretical constructs is challenging in every field of science. However, it is especially problematic in more complex disciplines. Without question, measurement is one of the criticized facets of social and behavioral studies. Researchers in the natural sciences commonly roll their eyes at the scales and instruments that are administered in fields such as psychology and education. Even social and behavioral researchers are often openly critical of the measures in their fields (e.g., Mitchell, 1999; Fried and Flake, 2018). We will not attempt to review the numerous shortcomings of social and behavioral measures that have been explicated in the literature. Instead, our focus is on why measurement is an intractable problem in the study of complex phenomena.

### The Problem of Generality

Many researchers (e.g., Mitchell, 1999; Finkelstein, 2005) have suggested that one of the principal reasons why measures are bad in the social and behavioral sciences is sketchy concepts and theories. Theoretical constructs in fields such as psychology and sociology lack the definitiveness needed for the development of valid instruments and scales (e.g., Meehl, 1978).

Scientific measurement is not a simple mechanical procedure of assigning numbers or symbols to attributes or events. The process generally begins with theory; theory determines to a large degree what is measured and how it is measured (e.g., Borsboom et al., 2004; Bringmann and Eronen, 2016). Scientists attempt to develop theories that are parsimonious and that have scope. This requires the development of constructs that are general and that can represent a broad array of attributes, processes, states, entities, or events. However, when the phenomena are complex, the instances or members of a construct are often highly diverse. In order to accommodate this variability, the theoretical constructs have to be defined abstractly and vaguely. It is these qualities that allow constructs to be more inclusive and general and applicable to a broader set of instances and contexts. As Zeller and Carmines (1980, p. 3) observed, “…abstract concepts can only be approximated by empirical indicants. Indeed, it is the very vagueness, complexity, and suggestiveness of concepts that allow them to be empirically referenced with varying degrees of success at different times and places.”

However, these qualities also contribute to seemingly never-ending debates about how to define theoretical constructs. For example, social and behavioral scientists have argued for decades about how to best define constructs such as “community,” “social class,” and “reward.”

More significantly, the lack of definitiveness of theoretical constructs of complex phenomena allows greater leeway in how they are operationalized. Because of their scope, none of the operationalizations fully capture all the facets and instances of the constructs (Zeller and Carmines, 1980). Moreover, theoretical constructs are often defined so abstractly and loosely that they are inclusive of properties, processes, and entities that they are not intended to represent. Thus, theoretical constructs that are supposedly distinct are commonly overlapping and redundant with one another, which leads to seemingly never ending debates about whether and how constructs are different from one another.

Finally, general constructs are often defined so abstractly and vaguely that they cannot possibly be precisely scaled. Because many constructs amount to loose configurations of attributes, beliefs, states, behaviors, and processes that are lacking a quantitative or quantifiable structure (Mitchell, 1997, 1999), it is impossible to determine exactly what increases and decreases in the constructs mean or entail. These, of course, are the fundamental issues of validity that bedevil measurement in the social and behavioral sciences. When constructs are defined abstractly to be inclusive of a broad array of properties, it is almost impossible to develop measures that correspond to the intended concept and only the intended concept.

Many researchers are aware of the disconnect between the constructs of complex theories and the measures that are used to measure them. To increase uniformity and precision, some resort to narrowing their theories and defining their constructs in more operational terms. This may seem like an instance of putting the cart before the horse but tying theories more closely to scales and instruments is frequently necessary to deal with the measurement morass pervading the study of complex phenomena. Applied quantitative models in the social and behavioral sciences are routinely limited to variables that have well established and tightly defined operationalizations.

The problem with defining constructs more narrowly, of course, is that it reduces the scope of the theory. This is the terrible tradeoff between generality and precision that researchers commonly have to make in the development of theories of complex topics (Sanbonmatsu and Johnston, 2019). Psychological scientists create basic theories to facilitate the explanation and understanding of a broad array of phenomena. However, the theories invariably lack the specificity and definition needed for precise measurement and prediction. While narrowing the constructs allows for the development of more valid and precise measures, it comes at the expense of the generality of the theory.

This tradeoff is manifested in the literature on self-esteem. The great strength of the construct is its scope and explanatory value. Studies of self-esteem help to explain, in part, a wide variety of important outcomes ranging from resistance to persuasion to occupational and educational success. However, the breadth of the construct is also its weakness. Because self-esteem is so loosely defined, there are a multitude of different scales used to measure it, many of which are weakly correlated or uncorrelated with one another (Wells and Marwell, 1976; Wylie, 1979). Naturally, this has led to varied and sometimes conflicting findings which have limited the ability of researchers to draw coherent theoretical conclusions about the causes and effects of self-esteem. What appears clear is that, with the possible exception of happiness, self-esteem is not a major predictor of anything (Baumeister et al., 2003), a pattern that is common for constructs that are broad and amorphous. Finally, there are numerous constructs, such as narcissism and extraversion, that co-vary significantly with self-esteem in the prediction of important outcomes. Not surprisingly, there are long standing disagreements about how to define and measure self-esteem and about how self-esteem differs from other personality variables.

In our view, the difficulties of measuring general constructs of complex social and behavioral phenomena are largely intractable. To accurately represent the variability characterizing broad and often diverse categories of complex entities, properties, and events, general constructs must be abstract and vague to the point that the development of highly valid and precise instruments is precluded. To develop better measures, the scope of the constructs of a theory or model must be limited.

### Internal Constructs and Processes

A second way in which theory contributes to inadequate measurement in psychology is the postulation of cognitive and affective processes, states, and constructs such as traits and attitudes that are not directly observable. As generations of researchers have pointed out, these constructs do not lend themselves to precise scaling (e.g., Mitchell, 1999; Blanton and Jaccard, 2006). Moreover, they are generally measured through self-report, which is fraught with problems such as social desirability bias and misremembering. While cognitive neuroscientists are working diligently to develop psychophysiological measures of mental processes, self-report remains by far the most common and best method to measure constructs such as perceptions, intentions, feelings, and traits.

However, the complexity of social and behavioral phenomena necessitates the inclusion of internal processes in the construction of psychological theories. Researchers have long realized that, to explain and predict behavior, they often must analyze both the stimulus situation and individuals’ unique processing of and responding to the stimulus situation. The measurement of mental states and constructs enables researchers to account for the idiosyncrasies of persons in their theories.

Moreover, because mental processes are generally the most proximal determinants of responding, they are often the best predictor. Human action is often shaped by innumerable events and developments that take place over a lengthy period of time. Researchers have learned that complex behaviors are generally much better predicted by internal constructs, such as traits, attitudes, and intentions, than by more temporally distal forces. The prevailing hope in psychology, of course, is that the greatest advancements in the field will be achieved through the study of the proximal psychophysiological processes underlying cognition, affect, and action. Thus, while the postulation of internal processes and constructs in psychological theories creates a myriad of measurement issues, it is essential for the explanation and prediction of behavior.

## The Effect of Complexity on Replication

Is there currently a crisis of confidence in psychological science reflecting an unprecedented level of doubt among practitioners about the reliability of research findings in the field? It would certainly appear that there is (Pashler and Wagenmakers, 2012, p. 528).

A presumed requirement of rigorous scientific inquiry is replication (e.g., Francis, 2012); research findings must be reproduced to ensure that they are reliable and, hence, a sound basis for scientific inference. Replication has been idealized as a cornerstone of science in every discipline from chemistry to psychology. However, in our view, substantial variability in the frequency and degree of reproduction of studies is to be expected across fields because of differences in the complexity of the study phenomena.

The belief in the sanctity of reproduction is based heavily on research in the physical sciences in which findings have been regularly reproduced because the study phenomena are often relatively simple. The causal relations that are investigated are commonly strong and invariant across context which makes reproduction more likely. However, as complexity increases, the connections between constructs tend to be weaker and more numerous and variable. Moreover, complex systems often mutate as a result of encounters with the broader environment. Thus, theory suggests that as the complexity of the topics increases, the uniformity of relations and effects across context and time should be lower. In particular, findings in psychology and other social and behavioral sciences should commonly be less robust and reproducible than findings in the physical sciences.

Consistent with theory, several systematic, large-scale replication projects have shown that the reproduction rates of published psychology studies are often low. For example, in the Open Science Collaboration (2015), only 36% of the replication studies reported statistically significant results whereas 97% of the original studies reported significant results. The effect sizes were half the magnitude of the original study effects. A replication of social science studies reported in *Nature* and *Science* from 2010 to 2015 (Camerer et al., 2018) generated significant findings similar to those of the original study in 62% of the replications, though the effect sizes were substantially lower. In the most recent Many Labs Reproducibility Project (Klein et al., 2018), 54% of the replications found a statistically significant effect in the same direction of the original finding. Most (75%) observed effects were smaller than those of the original study. Thus, while a large proportion of the studies were reproduced by these arduous and important replication projects, a similar large proportion of the studies were not reproduced. Moreover, the effect sizes observed in the replications were generally smaller.

The impact of complexity on the reproducibility of research findings is further implicated by data showing that the replication rates are lower in social psychology than in cognitive psychology (Open Science Collaboration, 2015). There are undoubtedly many factors that contribute to differences in reproducibility between these sub-disciplines, including the greater utilization of within-subjects designs and multiple trials in cognitive psychology. However, we would expect social psychological studies to be more difficult to replicate given the greater complexity of the phenomena that are studied. Researchers in the field generally investigate complex interpersonal and group processes and social behaviors and cognitions that are subject to substantial variation as a function of persons, culture, and time. The effects observed in social psychology studies are also commonly weaker (Open Science Collaboration, 2015), which further diminishes the likelihood of replication.

Finally, a related analysis of the replication data generated by the Open Science Collaboration (2015) by Van Bavel et al. (2016) suggests that the contextual sensitivity of findings in psychology affects their reproducibility. Correlations and effects that were perceived to be more readily influenced by culture, time, and place were less likely to be successfully replicated. Again, complex phenomena are moderated by a broader array of components or constructs, and, hence, are less consistent across contexts.

### Why Has There Not Been a Replication Crisis in Other Social and Behavioral Disciplines?

Our analysis of the effects of complexity on reproduction suggests that the difficulties in replicating research findings should be greatest in the social and behavioral disciplines that examine organizational, societal, and cultural processes because these phenomena are the most complex. While replication has become an important issue and topic of research in economics (e.g., Camerer et al., 2016; Berry et al., 2017; Clemens, 2017), there does not appear to have been a replication crisis in anthropology, sociology, and political science and allied fields such as management, and communication. Certainly none of these social and behavioral disciplines has endured the widespread hand wringing, finger pointing, self-loathing, and general angst that has beset psychology.

There are probably many reasons why there has not been an uproar about replication in most social and behavioral sciences. Fields such as sociology, political science, and economics make heavy use of data collected by government agencies and other organizations that are shared by and available to most investigators which serves to minimize misuse or misconduct. In addition, researchers in these fields are less likely to conduct experiments which have been the primary target of most replication efforts. However, we speculate that the main reason why the turmoil has been limited to psychological science is because research findings are less expected to replicate in other social and behavioral disciplines. Sociologists, political scientists, organizational and management scientists, and anthropologists may be more cognizant of how the behaviors and processes they investigate vary as a function of societal conditions, groups, and cultures. In addition, they may be more apt to recognize that changes in the world can radically alter the functioning of individuals, organizations, and communities. In contrast, many theoretically oriented psychologists are convinced that they are investigating “basic processes” that are largely consistent across context and enduring for all time. However, while many psychophysiological and cognitive studies examine relatively simple relations and effects that are often highly reproducible, the replication literature suggests that the topics of study in most domains of psychology are not so “basic” and invariant.

### Replicating Variable and Weak Effects

The assumption that research findings must be reproducible and reproduced is another belief that has been adopted from the natural sciences where the phenomena of study are simpler. However, this convention may not apply to more complex sciences such as psychology because of the variable and weak effects that are often studied.

Numerous analyses have shown that a principal contributor to replication failure in psychological science is the combination of random error and small sample sizes (e.g., Stanley and Spence, 2014; Loken and Gelman, 2017). There appears to be consensus that the majority of studies in psychology have been severely underpowered (e.g., Cohen, 1962; Maxwell, 2004; Fraley and Vazire, 2014), given the sizeable noise that surrounds responding in social and behavioral studies. Many scholars have called for substantial increases in the sample sizes of psychology studies to eliminate the publication of spurious chance findings due to a random error and inflated effect size estimates resulting from the premature stopping of data collection. While some of the variability can be reduced through better measures and more uniform procedures, we believe that much of the random error is inherent to studies of complex phenomena. As we discussed previously, many psychological constructs cannot be measured precisely or reliably. Moreover, research settings, procedures, and participants feature a multitude of moving parts that can impact behavioral responding. Consequently, high levels of noise may be characteristic of psychological research.

In line with the tenets of scientific determinism, we believe that, in principle, “exact” replication studies should obtain the same findings as the original study. However, many scholars have pointed out (e.g., Rosenthal, 1991; Fabrigar and Wegener, 2016) that “exact” replications in psychology are never exactly the same. Even when researchers diligently strive to duplicate the manipulations and measures of a study, there are inevitably variations in the interactions, participants, laboratories, institutions, and cultures. Moreover, even when the procedures are identical, they often vary in terms of how they are perceived by participants (Stroebe and Strack, 2014). While the phenomena studied in all fields are affected by context, the extraneous factors operating in studies of physical effects may generally be fewer in number and more readily controlled.

Finally, replications are often conducted months and years following the publication of a study and after momentous political, social, and environmental events have altered attitudes and the societal milieu. While these events may not change the effects of temperature on the physical state of water, they do influence many of the complex processes and behaviors that are studied in psychological science. Conceptual replications, of course, are even less likely to reproduce the findings of a study (e.g., Earp and Trafimow, 2015). As we discussed previously, many of the theoretical constructs studied in psychological research are abstractly and loosely defined. Consequently, operationalizations of the same construct are often very dissimilar from one another.

In keeping with arguments about the effects of context on reproduction, evidence indicates that one of the most fundamental reasons why replication rates in psychology are low is the hetereogeneity of study effect sizes (e.g., McShane et al., 2019). As Stanley et al. (2018, p. 1325) state, “Heterogeneity… makes it unlikely that the typical psychological study can be closely replicated when replication is defined as study-level null hypothesis significance testing…” The variability of particular relations and effects varies tremendously as a function of the complexity of the phenomena under investigation and scientific discipline. Because complex phenomena commonly change across context and time, a broader range of effect sizes may be generated by social and behavioral studies than by research in fields such as chemistry.

While an average or typical effect size can be calculated for a set of studies, any notions that there is a singular universal “true effect” or “true correlation” characterizing complex phenomena across studies conducted in different settings and times are generally wishful thinking. The expectation that the same mathematical relation will be uncovered by each and every replication study in psychology is at odds with the empirical data. Thus, Patil et al. (2016, p. 540) point out that when replication studies are conducted, researchers should not expect “the same numbers will result for a host of reasons including both natural variability and changes in the sample population, methods, or analysis techniques.”

While there are few, if any, broad studies that have systematically compared effect sizes across disciplines (for comparisons between psychology and other disciplines, see Hedges, 1987; Ferguson, 2009), research shows that the relations studied in psychological research are commonly weak (e.g., Hemphill, 2003; Schafer and Schwarz, 2019) and, hence, less reproducible (Ioannidis, 2005). When the finding of a published study is weak to the point that it just meets the threshold for “significance,” a sizeable portion of the replication studies will be non-significant. If reproduction failure is defined at all in terms of failing to attain statistical significance, this is evidence that the original findings were false and a Type I error. However, the negative results could simply be the consequence of conditions or contexts that are less conducive to the effect.

A number of scholars have pointed out that when even replication studies fail to achieve statistical significance, they typically show the same patterns or directional trends and, thus, to a large degree “reproduce” the original findings. For example, an analysis of the studies of the first Many Labs Reproducibility Project (2014) by Patil et al. (2016) showed that 77% of the replication effect sizes reported were within a 95% prediction interval (one-way) based on the original effect size. All of this has led to informative discussion and some disagreement about what constitutes reproduction (e.g., Maxwell et al., 2015; Open Science Collaboration, 2015; Patil et al., 2016). Gelman (2018) argues that we should not even characterize replications as successes or failures.

If a study finding falls squarely in the middle of the distribution of possible effect sizes, half of the replication findings might exhibit stronger correlations or effects. However, this is not the pattern that is typically observed across replication studies. The majority of the relations observed in replication studies are weaker than those of the originally published research. As we discuss shortly, questionable research practices including the “publication bias” foster the reporting of studies that are hard to reproduce.

### Questionable Research Practices

The high frequency of replication failure in psychological science is generally attributed to questionable research practices or outright fraud rather than the complexity of the study phenomena. Scholars generally believe that studies are reproduced less often in disciplines such as psychology and medicine than in the natural sciences because misconduct is more commonplace.

Research has uncovered a number of specific practices such as rounding down *p*-values and claiming to have predicted an unexpected result that can lead to erroneous conclusions and the publication of unreproducible findings. Experimenter bias and demand effects may also contribute to the unwarranted confirmation of the hypothesis under investigation (e.g., Rosenthal and Rosnow, 2009; Klein et al., 2012). More broadly, scholars have suggested that there is a “publication bias” in favor of positive results (e.g., Ferguson and Heene, 2012) that contributes to the “file drawer problem” (e.g., Rosenthal, 1979; Franco et al., 2014). Together, these contribute to inflated estimates of the size of relations or effects in psychology studies. This is evidenced not only by failures to replicate but by analyses showing that there are significantly more reports of studies that reject the null hypothesis than is consistent with a power or Bayesian analysis of the body of work (e.g., Renkewitz et al., 2011; Etz and Vandekerckhove, 2016).

Inappropriate research practices are certainly a major problem in psychological science. For example, a survey study by Fiedler and Schwarz (2016) showed that while data fabrication is rare, a sizeable percentage of psychologists admit to specific practices such as deciding whether to continue data collection after testing the significance of the results (over 40%) at least once in their careers. However, there is little direct evidence showing that inappropriate research practices are more common in psychology than in other fields. For example, there are no studies showing that psychologists are more likely than other scientists to treat *post-hoc* hypotheses as *a priori* hypotheses. The fact is that very few studies have systematically compared the prevalence of misconduct in psychology vs. other disciplines. Instead, scholars and journalists have assumed that clearly inappropriate research practices are more rampant in psychological science than in other fields because the frequency of reproduction in the field is low and effect size estimations are inflated. They have similarly assumed that the motivations and processes contributing to misconduct are more prevalent in psychological science.

However, it is silly to think that the incentives to engage in inappropriate practices are greater in psychology than in other disciplines. As we all know, the potential financial rewards that ostensibly motivate scientists to engage in misconduct are far lower in psychology than in fields such as chemistry and engineering. Any suggestions that greater scientific fame and stature can be achieved in psychology than in other fields are similarly ridiculous. Fox (1990) argues that, because the stakes are lower, data fabrication and falsification are less frequent in the social and behavioral sciences. Some scholars (Weinstein, 1979; Braxton and Hargens, 1996) have also suggested that the policing of professional behavior is higher in “low consensus” or “soft” sciences than in “high consensus” sciences, which would further diminish the inclination to fudge.

Finally, there are no data to suggest that psychologists have lower ethical standards, less training in correct practices, or lower knowledge of research pitfalls. If anything, psychologists are more likely than other scientists to be informed about research bias and misconduct because these are inherently fascinating and important behavioral topics that are relevant to core areas of the field, such as judgment and decision making, and moral psychology. We speculate that one of the reasons why there has been an avalanche of studies on replication in psychological science is because it is such a rich research topic. We would further argue that psychologists are much better equipped than scientists and scholars in other fields to understand inferential errors in science and violations of research norms.

While there is little direct evidence showing that psychologists are generally more guilty of misconduct than other scientists, there are two specific methodological practices affecting reproducibility that appear to be more prevalent in psychology than in many other fields. Importantly, both of these problems arise, in part, because of the complexity of social and behavioral phenomena.

As we discussed previously, one contributor to replication failures in psychology has been underpowered studies (e.g., Cohen, 1962). However, the samples of many studies have been too small largely because of the inordinate noise that is inherent to complex psychological phenomena. Thus, it is complexity that has helped to make sampling practices in the field questionable. In our view, undersized samples in past studies should not be regarded as misconduct because most researchers were abiding by the prevailing norms for sampling.

The publication bias contributing to inflated effect sizes and replication failures (e.g., Ferguson and Heene, 2012) also appears to be more problematic and pronounced in psychology than in the physical sciences. The prevailing norm in all scientific disciplines is to engage in a confirmatory search and publish positive findings (Fanelli, 2012; Fanelli et al., 2017). Non-relations in nature are infinite in number and are typically theoretically uninteresting. While the publication bias tends to be universal, its impact on reproducibility varies substantially across fields. Researchers in simpler disciplines generally get away with focusing on positive effects because they are more invariant and reproducible. In contrast, positive findings in psychology and other social and behavioral sciences are less likely to be reproduced because of the heterogeneity of the relations. Hence, the publication bias is far more problematic in complex fields.

As we discussed previously, the norm to seek and publish positive results also appears to be stronger in psychological science (Fanelli, 2010) because of the complexity of behavioral phenomena. Science often progresses through the initial demonstration of an effect followed by an examination of the boundary conditions. This is particularly necessary in complex disciplines where the causal relations are lacking in uniformity. Typically, psychologists set up a study in a way that maximizes the likelihood of demonstrating a hypothesized relation in a particular research context. This has been aptly pointed out by Fiedler (2011) who persuasively argued that researchers “select stimuli, task settings, favorable boundary conditions, dependent variables and independent variables, treatment levels, moderators, mediators, and multiple parameter settings in such a way that empirical phenomena become maximally visible and stable. In general, paradigms can be understood as conventional setups for producing idealized, inflated effects” (p. 163). Designing research that optimizes the chances of showing an effect often leads to the publishing of findings that are atypical and not reproducible across all contexts. However, as we suggested previously, it is impossible to construct a psychology study that is representative of the universe with respect to the settings, participants, and operationalizations because of the complexity of these variables. For example, in a study of persuasion, what message could be presented to participants that is representative of all possible communications? How would one choose a physical environment for a study that is representative of all the physical environments in the world? Instead, psychological scientists demonstrate a phenomenon using select samples of the important study components with the expectation that subsequent research will investigate the conditions in which the effect occurs and does not occur. While the pronounced bias toward positive results in complex fields such as psychology contributes to inflated initial effect size estimates, the approach is necessary given the uninformativeness of most null findings.

### The Importance of Replication Studies

The replication crisis in psychology has advanced the discipline in numerous ways. The documentation and analysis of reproduction failures has led to the implementation of new research norms and procedures, many of which are vital to the integrity and future of the field (e.g., Asendorpf et al., 2013; Funder et al., 2014; Nelson et al., 2018). One of the most important developments in psychology has been the sanctioning of replication studies and negative results. Researchers are conducting and publishing replication studies to restore confidence in the field and to advance theory. Most scientists believe that the primary purpose of replication studies is to determine whether a finding is real and duplicable. However, when the study phenomena are complex, any expectations of exact quantitative reproduction across studies are unrealistic and contrary to the data on the hetereogeneity of replication findings (e.g., Patil et al., 2016; Stanley et al., 2018). Following McShane et al. (2019), we believe that one of the primary aims of replication studies should be to help profile a correlation or effect. Because of the complexity of most social and behavioral topics, it is important to examine the variability and robustness of the test relation (Stanley and Spence, 2014) within a range of contexts. Replication studies and meta-analyses can also provide a better estimate of the typical effect size and diminish the publication bias (Stanley et al., 2018). Finally, follow-up research may help to uncover important moderators of a correlation or effect. Because of the importance of examining the variability of effect sizes, it may be far more useful to conduct multiple studies with reasonably sized samples in different labs and contexts than conducting a single study with a mega-sample in a particular lab.

### What Do Replication Studies Say About Generalization?

Psychological science has been understandably preoccupied with replication for the past decade. However, the broader and more important topic that has been overlooked in all of the hubbub is the generality of research findings in the field. While replication is important, it is just part of the more central issue of generalization.

Replication studies in complex fields can be seen as tests of whether the results of a study generalize to highly similar conditions or contexts using the same procedures. The empirical data starkly illuminate the formidable beast that social and behavioral scientists attempt to capture in their research. The variability characterizing complex phenomena is so great that study findings often do not generalize to even highly similar conditions.

New discoveries in psychological science are often touted for their importance and potential applications. However, when the topic is complex, the studies that follow commonly uncover boundary conditions that severely limit the scope of the findings. Moreover, there is typically an array of unspoken moderators that are not explicit in the conceptualization that further diminish the generality of the relation or effect. In the end, the findings and applications are often proven to be much narrower than researchers initially hoped and believed.

### The Most Straightforward Conclusion

The belief of many scientists and journalists has been that questionable practices and errant researchers are responsible for the reproduction crisis in psychology. Many research findings have been assumed to be fake or false because they have not been consistently reproduced in follow up studies. Paralleling this, some researchers have been accused of misconduct and incompetence because their studies have not been reliably duplicated.

While some published findings are spurious, there is a more straightforward explanation for the numerous replication failures in psychological science that does not entail the wholesale dismissal of the data. Following Stanley et al. (2018), and other researchers, we believe that divergent findings are an indication of the variability of the study effects and correlations. As McShane et al. (2019, p. 102) state: “heterogeneity is not only the norm but also cannot be avoided in psychological research – even if every effort is taken to eliminate it.” More broadly, we believe that replication failures reflect the complexity and limited reproducibility of psychological effects. This conclusion is supported not only by the data but also by theory. Social and behavioral phenomena are less general, more mutable, and weaker than those investigated in the physical sciences. As a consequence, the correlations and effects observed in studies are much less consistent across context. In our view, the conclusion that most replication failures are due to fake or false findings is dubious because it implies that the variability in results will disappear when more ethical and rigorous practices are put into place.

Replication has long been regarded as a cornerstone of research. However, the lofty benchmarks that prevail in science have been based largely on studies of simpler topics. The research on replication in psychology provides strong empirical evidence that the standards that are applied in science should vary as a function of the complexity of the study phenomena and discipline.

## Why Social and Behavioral Science Lacks Rigor

“Because of the success of science, there is a kind of a pseudo-science. Social science is an example of a science which is not a science. They follow the forms. You gather data, you do so and so and so forth, but they do not get any laws, they have not found out anything. They have not got anywhere – yet. Maybe someday they will, but it’s not very well developed… Now, I might be quite wrong. Maybe they do know all these things. But I do not think I’m wrong. See, I have the advantage of having found out how hard it is to get to really know something, how careful you have to be about checking your experiments, how easy it is to make mistakes and fool yourself. I know what it means to know something. And therefore, I see how they get their information. And I cannot believe that they know when they have not done the work necessary, they have not done the checks necessary, they have not done the care necessary. I have a great suspicion that they do not know…” (Nobel Laureate Richard Feynman quoted in Johnson, 2009).

The replication crisis in science has triggered a renewed focus on rigor in research. Like most broad behavioral constructs, “rigor” has been defined in different ways. The National Institutes of Health (2015) defines scientific rigor as “the strict application of the scientific method to ensure robust and unbiased experimental design, methodology, analysis, interpretation and reporting of results. This includes full transparency in reporting experimental details so that others may reproduce and extend the findings.” Some researchers define rigor more abstractly as “theoretical or experimental approaches undertaken in a way that enhances confidence in the veracity of their findings, with veracity defined as truth or accuracy” (Casadevall and Fang, 2016). Both of these definitions focus on methods and approaches that increase the validity of scientific findings. However, “rigor” is also commonly defined more behaviorally as being careful, exact, and precise and adhering to strict standards of research.

Regardless of how the term is defined, there seems to be agreement that the social and behavioral sciences are less rigorous than the natural sciences. As the quote by the eminent physicist Richard Feynman suggested, many people believe that fields such as psychology and sociology are “pseudo sciences” that “have not done the checks necessary” and “have not done the care necessary.” We wish we could disagree with the critics and point smartly to data in support of the contrary. However, objective indices, such as reproduction rates and reliability, seem to verify that psychological science is generally lacking in rigor.

Paralleling other methodological issues, we believe that the limited rigor in fields such as psychology is partly attributable to logistical, financial, institutional, and ethical constraints and is largely attributable to the research phenomena. As we discuss shortly, the complexity of social and behavioral topics often precludes the implementation of highly rigorous methods of study. While the practices fall short of the standards of the physical sciences, we believe that they are efficient given the inherent difficulties of conceptualizing and studying complex phenomena.

### Modest Goals

Research on motivation suggests that individual performance and achievement often begins with goal setting. People set goals for themselves that are lofty or lowly or in between. Research (Locke and Latham, 1990) has shown that the types of goals and expectations that people begin with affects the success that they attain. Persons who set easy goals for themselves tend to accomplish less than persons with higher aspirations and expectations.

We believe that the lack of rigor in the social and behavioral sciences begins with the modest goals that guide research. For decades, the standard approach taken by most studies in psychology has been null hypothesis testing. Research has sought to provide evidence that the null hypothesis is not true, and there is a relation between variables or a difference between conditions. More broadly, psychological science has been largely content to identify causes and effects without specifying the exact mathematical relation between them. As we shall discuss, basic studies in psychology rarely test parameter estimates. That is, they infrequently attempt to verify specific estimations of the strength of a relation or the magnitude of an effect.

Null hypothesis testing has been rightfully criticized on a number of grounds (e.g., Meehl, 1978; Gigerenzer, 1998). However, one of the biggest problems is that it encourages sloppiness (Szucs and Ioannidis, 2017). Null hypothesis testing aimed at demonstrating a mere difference or relation disincentivizes researchers to be precise and careful.

## Building on Weakness

Henry Ford, it is said, commissioned a survey of the car scrap yards of America to find out if there were parts of the Model T Ford, which never failed. His inspectors came back with reports of almost every kind of breakdown: axles, brakes, pistons – all were liable to go wrong. But, they drew attention to one notable exception, the kingpins of the scrapped cars invariably had years of life left in them. With ruthless logic, Ford concluded that the kingpins on the Model T were too good for their job and ordered that in the future they should be made to an inferior specification (Humphrey, 1976, p. 303).

Although the accuracy of this tale about the business acumen of Henry Ford has been vigorously disputed (e.g., Hawks, 2005), a gem of an idea is presented that is relevant to many industries and endeavors including science. The output or productivity of many systems is limited by the weakest or worst performing parts. In some instances, expenditures may be decreased and efficiency may be increased by reducing the quality of the stronger or better performing components. Similarly, there is little utility in increasing the quality of a component if the general output is capped by the weakness of other parts.

Research studies consist of many different “parts” such as the sample, design, procedures, manipulations, measures, and analyses. As we reviewed previously, the rigor of many of these parts is compromised by the complexity of social and behavioral topics. Psychological scientists are generally aware of the weaknesses of various components of their studies. Moreover, many know that the generalizability of their research is severely restricted by the lack of representativeness of the study components and the variability of the research phenomena. We speculate that psychologists and other social and behavioral scientists are practical and efficient. They realize that the output of their research, that is, the conclusions that can be drawn, are limited by the weakest facets of their studies. Cognizant of these shortcomings, they efficiently minimize investing excessive time, effort, and funding on important components of their research. That is, they build some elements of their studies to an “inferior specification.” For example, why bother developing a precisely calibrated experimental manipulation when the scales of the measurement instruments are ordinal? Why expend resources on getting an exacting estimate of the sample population when the findings will not generalize across contexts?

When this efficiency is combined with the modest goal of showing that there is a relation between variables or a difference between conditions in null hypothesis testing, the rigor of research invariably suffers. The collective effect of these disincentives is to generally diminish the carefulness and precision of studies in the field. However, limiting rigor in this way is often the most sensible way of conducting research in psychological science. In the end, the most that researchers are typically able to conclude theoretically from their studies is that there is a relation or difference. Consequently, psychologists often do just enough to demonstrate a relation or difference.

Obviously, psychological science does not always work in this way. Research components sometimes have to be overbuilt to compensate for key weaknesses of a study. In some instances, the manipulations are so weak or the measures are so insensitive that the standards for other components have to be raised just to show a relation or effect. For example, current neuroimaging techniques are so noisy, crude, and expensive that study manipulations and procedures often have to be just right to register an effect on the measurements of the magnet.

### Easy Solutions

Some readers might conclude that the general lack of rigor in psychology can be readily resolved by raising standards in the field. Rather than merely showing a difference or effect in a study, psychologists should test the parameter estimates of their theories. The problem with this solution, of course, is that most basic theories in psychological science are qualitative. Hence, null hypothesis testing is perfectly suited for the vague predictions that are afforded by most basic theories in the field. This suggests another easy solution to the modest goals that disincentivize rigor in psychology: researchers should develop better theories. However, as Sanbonmatsu and Johnston (2019) point out, most basic theories in social and behavioral science are necessarily qualitative and vague because of the variability of the causal relations that are studied.

The mandate to present effect sizes in research reports (e.g., American Psychological Association, 2001) may be helping to improve the rigor of psychological studies. Although Cohen (1994, p. 1001) advised against looking “for a magical alternative to null hypothesis testing,” an increased focus on the size of relations or effects may incentivize researchers to upgrade their manipulations and measures. The development and testing of quantitative models may also raise the methodological standards of psychological research. Rigor matters more in tests of the predictiveness of applied models because the data serve as the basis for parameter estimation. Nevertheless, the rigor of psychological science will always be lower than that of fields such as physics and chemistry because of the complexity of the study phenomena. Complexity diminishes rigor by creating chronic problems in sampling, measurement, treatment, and reproduction. Complexity further limits rigor in psychology by diminishing the specificity of the hypotheses that can be tested and the conclusions that can be drawn and by disincentivizing researchers to be precise in all of the components of their studies.

We believe that if human behavior were simpler phenomena characterized by greater uniformity in causal relations across time and context, psychological science would be a very different enterprise. The rigor of research in the field undoubtedly would be an order of magnitude higher. Unfortunately, the failure to recognize the inherent limitations imposed by the complexity of behavioral phenomena and the continual pressure to achieve unrealistic scientific standards has been a continual source of criticism, self-doubt, and turmoil in psychology.

## Some Topics Are Harder to Study Than Others

Science is characterized by greater methodological diversity than ever before. As fields have progressed, the array of designs, analyses, and approaches used in research has grown tremendously. What has been made evident in recent decades is that there is no one correct way of practicing science. Different methods are needed to investigate different topics and to achieve different scientific aims. Research across disciplines has also taught us that some topics are much harder to study than others. The precise measures, exacting predictions, uniform experimental findings, and quantitative theories of classic work in physical science are simply not possible in most areas of scientific study. As a consequence, the standards that are used to evaluate methods, findings, and theories should be tethered to the complexity of the study topic. The characterization of psychology and other social and behavioral disciplines as “fake science” reflects complete ignorance of the numerous parameters that shape scientific practice and achievement.

The standards that guide social and behavioral research are not “lower,” less demanding, or less scientific than those of other disciplines. Rather, they are often different because of the complexity of the study phenomena and other constraints. For example, the singular accounts that sometimes characterize theory in other fields generally do not work in the social and behavioral sciences. As we discussed previously, explanation and prediction in disciplines, such as economics and psychology, frequently requires the investigation of multiple causes and interactions, sophisticated statistical tests to parse out the respective contributions, and modeling of the complex relations. Another example of different standards is in sampling where representativeness is a common issue that necessitates special techniques and large numbers.

In contrasting psychology with the natural sciences, we focused on differences and neglected the parallels between fields. However, it is important to recognize that the methodological challenges that we reviewed are not unique to the social and behavioral sciences. Difficulties in measurement, reproducibility, communication, and rigor characterize the study of complex phenomena in all disciplines. In the physical sciences, researchers commonly investigate topics that are difficult to conceptualize, measure, and predict. As Bringmann and Eronen (2016, p. 38) point out in their discussion of measurement practices in physics vs. psychology:

We believe that the differences are a matter of degree, and not as categorical as is often supposed. For example, although properties such as length or weight can be measured in a relatively direct and straightforward way, the same does not apply to phenomena such as the weak nuclear force or the background radiation of the universe. Such phenomena (which includes most phenomena studied in contemporary physics) can be measured only indirectly, and have no straightforward operationalizations (Kyburg, 1984).

Hedges (1987) provocative but limited analysis of replications in physics and psychology in physics and psychology “suggests that the results of physical experiments may not be strikingly more consistent than those of social or behavioral experiments.” Finally, physicists have increasingly turned to modeling and simulation because many of the phenomena they are investigating are not subject to reductionist analysis and representation by tight mathematical theories (Jogalekar, 2013). Thus, the differences in methods, procedures, findings, and theories that we have reviewed are more a function of the scientific endeavor and subject matter than discipline. As scientists in all fields take on phenomena of greater complexity, they are increasingly encountering the challenges that are inherent to the study of human behavior.

## Author Contributions

DS and JB conceptualized and wrote the paper. EC conducted the archival research that served as the groundwork for the paper. All authors contributed to the article and approved the submitted version.

### Conflict of Interest

The authors declare that the research was conducted in the absence of any commercial or financial relationships that could be construed as a potential conflict of interest.
